# Mapping frameworks for synthesizing qualitative evidence in health technology assessment

**DOI:** 10.1017/S0266462324000369

**Published:** 2024-11-13

**Authors:** Marilia Mastrocolla de Almeida Cardoso, Rafael Thomaz Marques, Juliana Machado-Rugolo, Lehana Thabane, Vilanice Alves de Araújo Püschel, Silke Anna Theresa Weber, Rosimary Terezinha Almeida, Graciela Paula do Nascimento Duque, Cristiane Cardoso De Paula, Luciane Cruz Lopes, Mariana Gabriel, Sybelle Drumond, Clarice Maria Rodrigues, Meredith Vanstone

**Affiliations:** 1 Hospital das Clinicas Botucatu, Botucatu, Brazil; 2 The Brazilian Centre for Evidence-based Healthcare – A JBI Centre of Excellence, Botucatu, SP, Brazil; 3 Faculty of Medicine, São Paulo State University, Botucatu, SP, Brazil; 4 Hospital das Clínicas da Faculdade de Medicina de Botucatu, Botucatu, SP, Brazil; 5 Health Research, Methods, Evidence and Impact, McMaster University, Hamilton, ON, Canada; 6 Biostatistics Unit, St Joseph’s Healthcare Hamilton, Hamilton, ON, Canada; 7 Faculty of Health Sciences, University of Johannesburg, Auckland Park, Gauteng, South Africa; 8 School of Nursing, University of São Paulo, SP, Brazil; 9 Biomedical Engineering Program COPPE, Federal University of Rio de Janeiro, Rio de Janeiro, RJ, Brazil; 10 Brazilian Hospital Services Company – EBSERH, Brazil; 11 Federal University of Santa Maria, Santa Maria, RS, Brazil; 12 Pharmaceutical Science Graduate Course, University of Sorocaba, Sorocaba, SP, Brazil; 13 Faculty of Dentistry, University of São Paulo, Brazil; 14 Rio de Janeiro State Murcoviscidosis Association, Brazil; 15 Fiocruz Strategic Studies Center, Brazil; 16 Hospital and health complex, Federal University of Rio de Janeiro, Rio de Janeiro, RJ, Brazil; 17 Department of Family Medicine, McMaster University, Hamilton, Ontario, Canada

**Keywords:** health technology assessment, qualitative research, review, evidence based health care

## Abstract

**Objectives:**

Health Technology Assessment (HTA) practitioners recognize the significance of qualitative methodologies that focus on how a technology is feasible, meaningfulness, acceptable, and equitable. This mapping aimed to delineate the frameworks employed to synthesize qualitative evidence and assess the quality of synthesis in HTA .

**Methods:**

Mapping was conducted using Medline, LILACS, CINAHL, Embase, Web of Science, Scopus, PsycINFO, Cochrane Library, JBI, and ScienceDirect databases. Gray literature searches included PROQUEST, Open Grey, Canadian Agency for Drugs and Technologies in Health’s Grey Matters, Google Scholar, and HTA agency websites. The inclusion criteria were centered on global qualitative evidence synthesis frameworks. The data are presented in the tables.

**Results:**

Of the 2054 articles, 31 were included, mostly from Europe. Guide was the type of document more cited, and most authors are from HTA agencies and universities. Incorporating both patient and family perspectives is the most cited reason for include qualitative evidence. Regardless of the framework or tool, SPICE was the main acronym, and RETREAT was preferred for approach selection. Thematic synthesis dominated analytic methods, and CASP was the primary quality appraisal tool. GRADE-CERQual graded evidence synthesis, with ENTREQ as the top reporting guidance. The GRADE evidence-to-decision framework was mentioned for recommendations.

**Conclusion:**

This mapping highlights the movement incorporate qualitative evidence in HTA employing specific frameworks. Despite the similarities among documents, most of them describe part of the process to synthesize qualitative evidence. Standardizing procedures to incorporate qualitative evidence into HTA can enhance decision-making. These findings offer essential considerations for HTA practice.

## Introduction

Health Technology Assessment (HTA) is a multidisciplinary process that uses explicit methods to determine the value of a health technology at different points in its lifecycle. Its objective is to inform decision-making to promote an equitable, efficient, and high-quality health system (1). Through this process, clinical guidelines or recommendation reports are developed to provide decision-makers with the best possible information. More recently, HTA practitioners have recognized that assessments are stronger when they integrate both quantitative and qualitative evidence (2–4). Qualitative evidence expands the evaluation “explaining why interventions are or are not effective from a person centered perspective, and address questions related to the usability, meaningfulness, feasibility and appropriateness of interventions.” (5, pg 12), which includes acceptability and equity (2).

According to Langlois et al (6) qualitative evidence highlights relevant aspects of the complex health decision-making process, such as the needs, values, perceptions, and experiences of stakeholders (policymakers, providers, communities and patients).

One way to strengthen the power of qualitative data is to combine qualitative primary studies and their findings. Through evidence synthesis of primary studies, it is possible to produce an stronger evidence that goes beyond the findings of each individual studies (7).

Globally, authoritative processes exist for the identifying, synthesizings and reporting quantitative data during HTA. Guidelines from HTA agencies present similar instructions for conducting a systematic review of effectiveness, as well as the process to determine the quality of evidence. This similarity allows homogenization, comparison among different contexts, standardization, and the ability to update previously generated information (8–10).

Outside the HTA field, similar approaches are published to describe how tosummarizate qualitative evidence (5;11;12), how to appraise of the quality of evidence, and methods to aggregate qualitative findings (13–23).

However, according to some authors, the role of qualitative research in HTA is still underway and is sometimes marginally understood.

According to Germeni et al. (24), instructions related to problems of acceptability and the subjective value of stakeholders, as well as contextual factors associated with the implementation of health innovations, have been largely disregarded. A recent study by Szabo et al. (25) demonstrated that although patient-based evidence was common in the submissions of the National Institute for Health and Care Excellence (NICE) and the Canada’s Drug Agency (CADTH), only 14/107 and 24/124 submissions, respectively, involved formal qualitative data collection.

Wang et al. (26) analyzed how qualitative evidence was employed in the guideline development process. The authors concluded that although most of the included guidelines were of high quality, there were limitations, such as the lack of involvement of any specialist in qualitative research, lack of quality assessment of the synthesis of qualitative evidence, and lack of detail when reporting the level of quality of the evidence and its recommendations.

A paper exploring the implications of qualitative evidence synthesis in guideline, to overcome this challenge was published. The authors offer a guidance on the choice of qualitative evidence synthesis methodology in the context of guideline developers. Flemming et al. (3)

In the HTA field, some agencies have begun to include information on how to include qualitative evidence synthesis in the assessment process.

In 2019, the Scottish Intercollegiate Guidelines Network (SIGN) included qualitative summaries in its HTA processes to reduce the variance between practice and outcomes identifying individuals’ perspectives based on their lived experiences (11).

In 2020, the Decision Support Unit, an external assessment center based at the University of Sheffield and commissioned by the NICE provided research and training resources to support the Institute’s Center for Health Technology Evaluation Programs, summarizing methodological developments that occurrefrom 2012 to 2020 by examining and critiquing existing mentions of qualitative evidence in PMG9 (Guide to the Methods of Technology Appraisal) and other relevant NICE methods (2).

Another initiative was developed by the independent Institute for Quality and Efficiency in Health Care from Germany, which presented a document called “General Methods” to guide the sequence of the individual steps in producing the work results in the HTA field and included qualitative evidence as a topic. (27)

Therefore, despite the relevance of the findings from qualitative evidence synthesis, there is limited guidance on how to assess and use this evidence in policy and practice (6).

In light of this challenge, a preliminary topic search was conducted using PROSPERO, Open Science Framework, MEDLINE, Cochrane Database of Systematic Reviews, and JBI Evidence Synthesis to identify systematic or scoping reviews that analyzed how the process of qualitative evidence synthesis has been proposed in field of HTA. Only one review of the literature was found, which was published in 1999. This review evaluated the use of qualitative methods for HTA (28). According to the authors, qualitative approaches and techniques have both strengths and limitations. The results demonstrate that qualitative research, conducted and analyzed correctly, can provide crucial information on the implementation and impact of health technologies (28). However, the authors did not examine the evidence synthesis process, considering what type of document guided this process and which instructions was provided.

This review aimed to map and describe the frameworks used to include, synthesize, and evaluate the quality of qualitative evidence in the HTA context, to identify the similarities and differences between approaches.

## Methods

The proposed mapping was consistent with the Preferred Reporting Items for Systematic Reviews and Meta-Analyses Extension for Scoping Reviews (PRISMA-ScR) (29;30). The protocol was registered at OSF number https://doi.org/10.17605/OSF.IO/P65FC, and was previously published (32).

## Review question(s)

What documents address the frameworks for synthesizing qualitative evidence for applications in HTA?

What methodological processes are proposed for synthesizing qualitative evidence within an HTA process (e.g., objective, review question, synthesis process, data quality assessment, evidence grading, and form of presentation and incorporation of data in the HTA report)?

### Types of sources

This mapping includesd guidelines, reports, text and opinion papers, and other study designs for the proposed mapping.

### Search strategy

A search strategy aimed at locating published and unpublished documents up to June 2023, such as guidelines, reports, systematic reviews, texts, and opinion papers, was used. An initial limited search of MEDLINE (PubMed) and Embase was conducted to identify relevant articles. The text words contained in the titles and abstracts the relevant articles and the index terms used to describe the articles were used to develop a complete search strategy for Medline (Pubmed), LILACS (BVS), CINAHL (EBSCO), Embase (Elsevier), Web of Science, Scopus (Elsevier), Cochrane Library, JBI Database, and Science Direct. Grey literature was searched on the PROQUEST, Open Grey, CADTH’s Grey Matters, Google Scholar, and HTA agency websites. The search strategy was adapted for each information source, including all identified keywords and index terms (see Supplementary material I). The reference lists of the documents included in the review were screened for additional papers.

Articles published in English, French, Spanish, Portuguese, German, or Italian were included, corresponding with the team’s expertise. Time restrictions were not imposed.

## Inclusion criteria

The acronym used was the PCC (Population, Concept, and Context). The population consisted of eligible documents that provided frameworks for synthesizing qualitative evidence for any technology, audience, or context. In this review, the term “frameworks” is used to refer to supporting structures around which something can be built. Considering” Framework” as a tool to guide the developer through a sequence of steps to complete a procedure.” (33).

The concept of this review is the application of qualitative evidence synthesis (QES) for HTA. Evidence synthesis is the process of combining data from the included studies to conclude a body of evidence. This process involves synthesizing study characteristics and statistically synthesizing quantitative data or aggregating qualitative findings (34). The concept of HTA follows the World Health Organization (WHO) guidelines.

Systematic evaluation of the properties, effects, and/or impacts of health technologies and interventions. It -encompasses both the direct, intended consequences of technologies and interventions, as well as their indirect, unintended consequences. This approach is used to inform policy and decision-making in health care, particularly regarding how best to allocate limited funds to health interventions and technologies. Interdisciplinary groups conduct assessment using explicit analytical frameworks, drawing on clinical, epidemiological, health economic, and other information and methodologies. It may be applied to interventions, such as including a new medicine in a reimbursement scheme, rolling-out broad public health programs (such as immunization or screening for cancer), priority setting in health care, identifying health interventions that produce the greatest health gain, offering value for money, setting prices for medicines and other technologies based on their cost-effectiveness, and formulating clinical guidelines (35).

This review included documents published in any context.

### Study or source of evidence selection

Following the search, all identified records were collated and uploaded to EndNote 20/2020 (Clarivate Analytics, PA, USA), and duplicates were removed. Two independent reviewers screened titles and abstracts to assess the inclusion criteria. Potentially relevant papers were retrieved in full and evaluated in detail by two independent reviewers based on the inclusion criteria. Reasons for excluding full-text documents were recorded and reported in the scoping review. Any disagreements between the reviewers at any stage of the selection process were resolved through discussion or consultation with a third reviewer. The search results were reported in full in the final scoping review and presented as a PRISMA flow diagram (36).

### Data extraction

Data were extracted by two independent reviewers using a data extraction instrument developed by them (Appendix II). The data included specific details about the bibliographic characteristics of the documents (e.g., year, proponent, and type of document) and information related to the concept relevant to the review question, including, but not limited to, how the authors define QES, plan, conduct, and interpret the synthesis of qualitative evidence; the acronym to guide the elaboration of the review question; the selection of outcomes; the outcomes reported by the participants; the instrumental tools to assess the methodological quality of the studies; the methods of extracting, analyzing, and synthesizing the findings; how to grade the evidence(s); and the development of recommendations. Any reviewer disagreements were resolved through discussion. No author of any document was contacted to request missing or additional data.

### Data analysis and presentation

The evidence presented should respond directly to the review objective and question(s). The data are presented graphically and in tabular form. A narrative summary accompanies the tabulated, and charted results and describes how the results relate to the objectives and questions of the review (37).

Patients, HTA unit members, researchers with experience in the HTA process and methods, and researchers with expertise in qualitative evidence synthesis were invited to participate in the discussions. The experts had access to the results in advance to inform their insights and suggestions.

## Results

**Figure 1.**
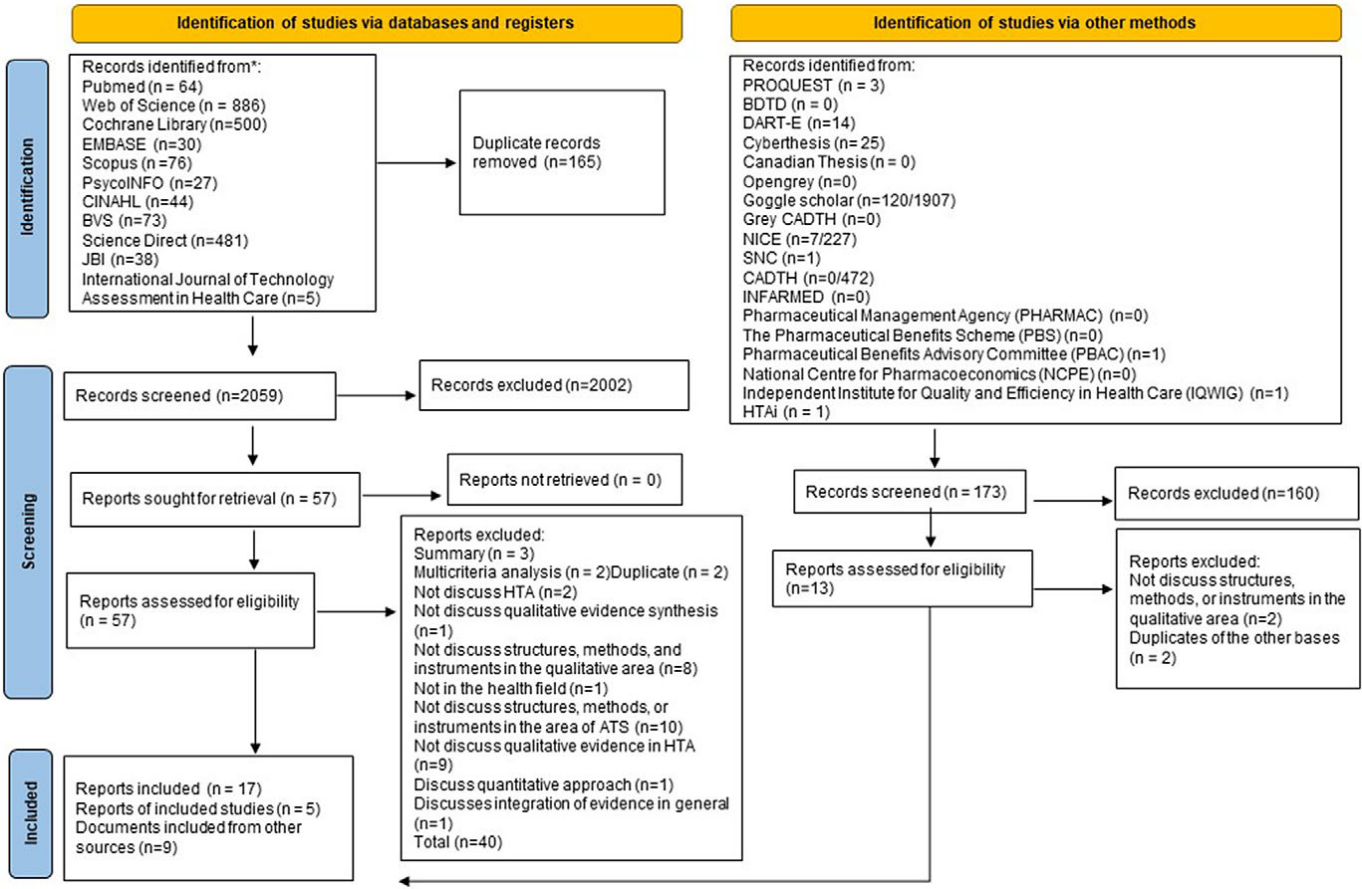
Document selection process, stages of title and summary reading, and full reading.

A total of 2,054 records were selected, with 165 duplicates. After reading the titles and abstracts, 1,997 documents were excluded. Fifty-seven documents were subjected to full-text reading, of which 40 did not meet the inclusion criteria, and 17 documents were considered eligible. In addition, nine records were added from other sources, such as HTA agencies or thesis databases, and five more records were added from the references, resulting in 31 documents (Figure 1).

### Characteristics of the documents

General characterization data were mapped regarding the document’s region of origin and authors, document format, year of publication, type of organization responsible for the document, and whether the responsible party was classified as a health technology agency (Table 1).

**Table 1.** Characteristics of the document based on the region, HTA agency, document type, and author’s context

Author, year	Region	HTA agency	Type of document	Author’s context
Barreto & Lewin, 2019 (38)	South America	No	Article	Foundation, Research Institute
Booth, 2020 (2)	Europe	No	Methodological document	University
Booth et al., 2016 (58)	Europe	No	Guide	University
Booth et al., 2018 (45)	Europe	No	Analytical article	University
Booth et al., 2018 (b) (45)	Europe	No	Review	University
Booth, 2017 (47)	Europe	No	Chapter/book	University
Campbell et al., 2019 (39)	North America and Europe	Yes	Review	University and Agency
Carmona et al., 2018 (46)	Europe	No	Review	University
Downe et al., 2019 (42)	Europe	No	Article Series	Organization, Research Institute or Health and University
Glenton et al., 2019 (43)	Europe	No	Article Series	Organization, Research Institute or Health and University
Healthcare Improvement Scotland, 2019 (57)	Europe	Yes	Guide	Organization
IQWiG, 2022 (27)	Europe	Yes	Methodological document	Agency
Langlois et al., 2018 (56)	Europe	No	Guide	Organization
Lewin et al., 2018 (20)	Europe	No	Article Series	Research Institute or Health e University
Lewin et al., 2015 (21)	Europe, Asia, Africa	No	Guide	Organization, Research Institute or Health and University
Lewin et al., 2019 (44)	Europe	No	Article Series	Organization, Research Institute or Health and University
Majid & Vanstone, 2018 (40)	North America	No	Review	University
NICE, 2012 (50)	Europe	Yes	Methodological document	Agency
NICE, 2013 (51)	Europe	Yes	Guide	Agency
NICE, 2014 (52)	Europe	Yes	Guide	Agency
NICE, 2022 (9)	Europe	Yes	Manual	Agency
Pfadenhauer et al., 2016 (65)	Europe	No	Guide	University
Pharmaceutical Benefits Advisory Committee, 2016 (54)	Europe	Yes	Guide	Agency
Ring et al., 2010 (59)	Europe	No	Guide	Organization
Ring et al., 2011 (49)	Europe	No	Methodological document	University and Organization
Santesso et al., 2021 (41)	North America	No	Guide	University
SBU, 2016 (55)	Europe	Yes	Guide	Agency
Scottish Intercollegiate Guidelines Network, 2019 (53)	Europe	Yes	Guide	Agency
Scottish Medicines Consortium, 2022 (11)	Europe	Yes	Guide	Agency
Steigenberger et al., 2021 (48)	Europe	No	Chapter/book	Health Institute and University
WHO, 2021 (60)	Europe	No	Guide	Organization

**Figure 2.**
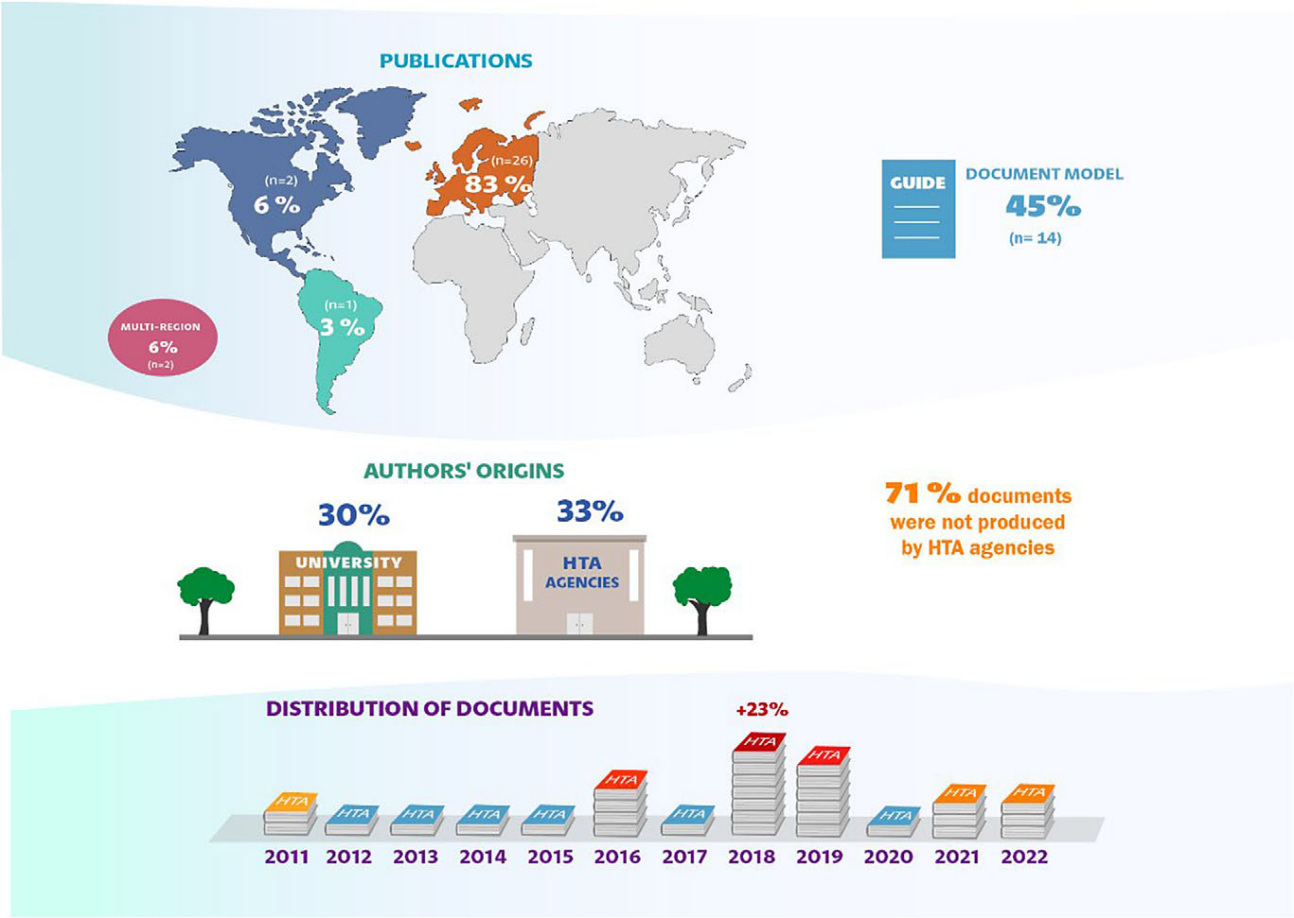
Characterization of the documents.

The synthesis of the data can be accessed in Figure 2.

Regarding the origin of the documents, there is a predominance of publications from Europa (n = 25, 81 percent), especially the United Kingdom, Sweden, and Germany. South America (38), multiple-regions (21), and North America and Europe (39) were each represented by one document. North America alone was represented by two documents (40;41).

Almost half of the documents were published as “guides” representing 45 percent (n = 14). Articles (series) (42–44) and reviews (39;40;45;46) were represented by four publications each. We also found two book chapters (47;48), and four methodological documents (2;27, 49;50). One document was titled a manual and the other an article (not a series) (9;38).

The data on the authors’ origins demonstrated that HTA agencies and universities are the places with the highest concentration, with 10 (33 percent) and nine (30 percent) documents, respectively. Universities in the United Kingdom and Canada were responsible for most of the publications. Nine documents were produced by two or more institutions (e.g. Research Institute and Foundation). (20;21;37;38;41–43;45;46). Four documents were published by organizations such as the WHO (55;59), the United Kingdom’s National Health Service (NHS) (58), and NHS Scotland (56).

Notably 22 (71percent) documents were not produced by the HTA agencies. Among those produced by agencies are the NICE, England (7;47–49), Scottish Medicines Consortium (SMC) (52), Pharmaceutical Benefits Advisory Committee (PBAC - Australia) (53), Swedish Agency for Health Technology Assessment and Assessment of Social Services (SBU) (54), and Institute for Quality and Efficiency in Health Care (IQWIG - Germany) (27).

The publication year of the documents was also mapped, showing an increase in publications in 2018, represented by seven documents, accounting for 23 percent. Eleven documents were published before 2018 (2011–2017), and 13 were published after 2018 (2019–2022). Documents published in 2010 or earlier were not identified.

### Specific data

The specific data addressed the second question of the mapping, “What methodological processes are proposed for synthesizing qualitative evidence within an HTA process (e.g., objective, review question, synthesis process, data quality assessment, evidence grading, and form of presentation and incorporation of data in the HTA report)?

The data presented in Table 2 address the rationale for including qualitative data, as well as proposals for structure, methods, tools, or specific instruments for using this type of data in the field of HTA.

**Table 2.** Specific data according to the concept, objective to include qualitative data, tools, framework, instrument, and checklist

Author, year	Concept	The objective to include qualitative data	Tools, framework, instrument, checklist, etc
Barreto & Lewin, 2019 (38)	*Qualitative evidence deals with empirical data extracted from research that uses qualitative data collection and analysis methods. Qualitative evidence includes evidence that emerges from individual primary qualitative studies as well as evidence from the findings of qualitative evidence syntheses (sometimes called Qualitative Evidence Synthesis to Inform Health Policy from Systematic Reviews of Primary Qualitative Studies – a form of secondary research).*	Opinions, the experiences and interests of the stakeholders	Assess confidence of findings - GRADE- -CERQual - Confidence in the Evidence from Reviews of Qualitative research
Booth, 2020 (2)	*an approach for synthesizing the findings from multiple primary qualitative studies”, “bring together evidence from multiple studies, thus providing richer data than a single study can”. “identify patterns in the data, explore similarities and differences across settings, lead to a new interpretive model or framework, and contribute broadly to a field of research*	View and opinion of patients, clinicians, family members and caregivers	Assess confidence of findings - GRADE- -CERQual - Confidence in the Evidence from Reviews of Qualitative research. Critical evaluation: CASP tool Cochrane qualitative checklist JBI checklist Cabinet Office checklist for social research
Booth et al., 2016 (58)	*A qualitative evidence synthesis can be used to explore important qualitative aspects of any HTA or SR decision-problem including whether a complex technology is acceptable, the lived experience of those with the target condition and issues relating to the implementation of the complex technology in context.*	Patient-centered approach	Framework - RETREAT (Research question, Epistemology, Time/Timeframe, Resources, Expertise, Audience & Purpose, Type of Data) framework.
Booth et al., 2018 (45)	NI	Patient-centered approach	Framework - RETREAT (Research question, Epistemology, Time/Timeframe, Resources, Expertise, Audience & Purpose, Type of Data) framework.
Booth et al., 2018 (b) (45)	*Provide evidence for diverse questions beyond those that typically relate to the feasibility and acceptability of complex interventions. QES can potentially provide rich data relating to the context of interventions, policies or conditions and the lived experiences, views and beliefs of those involved.*	Social perspective	Acronym - PerSPEcTiF (Perspective, Setting, Phenomenon of interest/problem, Environment (optional Comparison), Time/timing Findings)
Booth, 2017 (47)	*Vehicle for presenting patients’ attitudes, beliefs and feelings as originally captured by individual qualitative research studies. By aggregating or integrating views from multiple studies, rather than a single study, the science of systematic reviews takes steps to protect against allowing findings from an isolated study to overly influence our understanding or even to lead us to omit important perspectives.*	Patient perspective and participation	Framework -RETREAT (Research question, Epistemology, Time/Timeframe, Resources, Expertise, Audience & Purpose, Type of Data) framework e Garside (2008)
Campbell et al., 2019 (39)	*Methods used to search, select, and analyze findings from a set of primary qualitative research studies that relate to a specific topic or focus to arrive at new or enhanced understanding about the phenomenon under study*	The feasibility and acceptability of the intervention, the value of the outcomes for health services, and the impact of interventions on equity and human rights	Present qualitative synthesis: Narrative Summary, Thematic analysis, Metanarrative, Framework Synthesis
Carmona et al., 2018 (46)	NI	Patient-centered approach	Acronym - PICO ou SPICE Assess confidence of findings - GRADE- -CERQual - Confidence in the Evidence from Reviews of Qualitative research Critical evaluation: modified CASP tool; the Cochrane manual (Chapter 20), the cabinet office Quality in Qualitative Evaluation tool, and the Joanna Briggs Institute tool. Present qualitative synthesis: narrative synthesis, meta-synthesis,“imported concepts,“meta-ethnography, meta-study,qualitative meta-summary and framework analysis. INTEGRATE-HTA project Report the synthesis of Qualitative research - Enhancing Transparency in Reporting the synthesis of Qualitative research (ENTREQ), eMERGe for reporting of meta-ethnographies Recommendation - GRADE evidence-to-decision (EtD) framework (effectiveness, resource use, acceptability, feasibility and equity);
Downe et al., 2019 (42)	*A systematic review of qualitative studies, also known as a qualitative evidence synthesis (QES), is an approach for synthesizing the findings from multiple primary qualitative studies.*	Implications for values, preferences, acceptability, feasibility, and equity	Critical evaluation: CASP tool modified; Recommendation - GRADE evidence-to-decision (EtD) framework (effectiveness, resource use, acceptability, feasibility and equity); Assess confidence of findings - GRADE- -CERQual - Confidence in the Evidence from Reviews of Qualitative research
Glenton et al., 2019 (43)	*Systematic reviews of qualitative research, also known as qualitative evidence syntheses (QES)*	Acceptability, feasibility, and equity for service users and health workers	Recommendation - GRADE evidence-to-decision (EtD) framework (effectiveness, resource use, acceptability, feasibility and equity); Assess confidence of findings - GRADE- -CERQual - Confidence in the Evidence from Reviews of Qualitative research
Healthcare Improvement Scotland, 2019 (57)	*QES is a process in which researchers systematically review and synthesise the evidence coming from individual qualitative studies on the same topic of interest to create new understanding by comparing and analysing concepts and findings.*	Patients’ experiences, behaviours and preferences	Framework - NHS Patient Experience Framework, the EUnetha coreModel, the Warwick Patient Experience Framework, Danish Centre for Health Technology Assessment HTA (DACHENTA) Handbook Acronym - SPICE - In conflict settings (S), what are citizens’ (P) views of using telemedicine (I), relative to standard care (C), in terms of barriers and facilitators (E) Critical evaluation: CASP tool Report the synthesis of Qualitative research: Quality of Reporting Tool (QuaRT) Present qualitative synthesis: framework synthesis narrative summary and synthesis thematic synthesis Best fit framework approach
IQWiG, 2022 (27)	*NI*	To examine subjective experience. To explore experiences and individual actions as well as to understand social reality	-CERQual - Confidence in the Evidence from Reviews of Qualitative research Critical evaluation: CASP tool
Langlois et al., 2018 (56)	*The contextualization and integration of evidence on a particular topic, including the findings of individual research studies. The process of synthesis is defined as the creation of something new from separate elements which can include pulling together findings from multiple studies to answer a defined research question. The findings of evidence syntheses are often described as more reliable and valid than the results of individual studies, especially when the primary research results are conflicting. Evidence syntheses help in the collation, appraisal and reporting of research evidence through the use of transparent scientific methods that are detailed and reported in advance and that will be reproducible by others. A synthesis can take the form of a systematic review – defined as a review of the literature that uses systematic, explicit and accountable methods – and may collate and integrate quantitative and/or qualitative results.*	Stakeholder perceptions and views on specific health system challenges and policy options.	Assess confidence of findings - GRADE- -CERQual - Confidence in the Evidence from Reviews of Qualitative research Acronym - SPICE - In conflict settings (S), what are citizens’ (P) views of using telemedicine (I), relative to standard care (C), in terms of barriers and facilitators (E) SPIDER - Among citizens in conflicts settings (S, P), how is using telemedicine (I), relative to standard care (D), viewed in terms of barriers and facilitators (E) based on qualitative research (R) Present qualitative synthesis: Meta-ethnography, Realist review or synthesis, Narrative review or synthesis, Thematic synthesis, Framework synthesis, “Best fit” framework synthesis. Recommendation - GRADE evidence-to-decision (EtD) framework (effectiveness, resource use, acceptability, feasibility and equity);
Lewin et al., 2018 (20)	NI	People’s perception and experience	Assess confidence of findings - GRADE- -CERQual - Confidence in the Evidence from Reviews of Qualitative research
Lewin et al., 2015 (21)	NI	The feasibility and acceptability of the intervention, to better understand the factors that may influence its implementation	Assess confidence of findings - GRADE- -CERQual - Confidence in the Evidence from Reviews of Qualitative research
Lewin et al., 2019 (44)	*systematic reviews of qualitative studies (also known as qualitative evidence syntheses (QES)) – an approach for synthesizing the findings from multiple primary qualitative studies. Like systematic reviews of the effectiveness of interventions, QES can provide key evidence for informing guideline recommendations and other decisions*	Important outcomes for stakeholders, implications of the intervention on values, preferences, acceptability, feasibility, and equity	Recommendation - GRADE evidence-to-decision (EtD) framework (effectiveness, resource use, acceptability, feasibility and equity); Assess confidence of findings - GRADE- -CERQual - Confidence in the Evidence from Reviews of Qualitative research
Majid & Vanstone, 2018 (40)	NI	Patient and public perspective	Critical evaluation: CASP, JBI, Popay, QF, Walsh, ETQS Report the synthesis of Qualitative research: COREQ, SRQR
NICE, 2012 (50)	*Qualitative research studies (such as interviews or focus groups) can be used to examine the views of the target populations.*	experiences and opinions of the client or professional opportunities and barriers to improvement variations in delivery and implementation for different groups, populations or settings barriers and facilitators that assist in implementation social context and construction and social representation of health and illness context background, from an observer’s point of view an explanation of associations between interventions and outcomes.	Acronym - PICO framework Present qualitative synthesis: thematic analysis ‘conceptual mapping’ grounded approach meta-ethnography Critical evaluation: own instrument
NICE, 2013 (51)	*methods used for summarising (comparing and contrasting) evidence into a clinically meaningful conclusion to answer a defined clinical question.*	Patient experience of acceptability for different types of treatments	Present qualitative synthesis: thematic analysis
NICE, 2014 (52)	NI	Patient experience	NA
NICE, 2022 (9)	NI	Values, preferences, acceptability, feasibility and equity. Patient experience and quality of life. View of caregivers and clinicians	Present qualitative synthesis: rapid review framework synthesis narrative summary and synthesis meta-synthesis thematic synthesis.
Pfadenhauer et al., 2016 (65)	NI	Facilitators and barriers to implementation	The Context and Implementation of Complex Interventions (CICI framework)
Pharmaceutical Benefits Advisory Committee, 2016 (54)	NI	Expert opinion to fill information gaps	Own method
Ring et al., 2010 (59)	*Synthesis of qualitative evidence is, by contrast, more exploratory and may seek to expand understanding of a phenomena or patient experience.*	Patients’ needs and preferences and experiences. Person-centered service	Acronym - Population, Intervention, Comparison, and Outcome (PICO) model which has been adapted for qualitative studies by including the phenomena of interest (P) and context (C) Critical evaluation: CASP tool; Present qualitative synthesis: grounded theory synthesis meta-ethnography meta-interpretation meta-study meta-summary qualitative cross-case analysis thematic synthesis.
Ring et al., 2011 (49)	*Synthesizing qualitative research is one mechanism for ensuring that patient/carer views and perspectives are incorporated into health service policy making and delivery.*	needs, preferences, and experiences of service users patient- centered approach	Present qualitative synthesis: grounded theory synthesis meta-ethnography meta-interpretation meta-study meta-summary qualitative cross-case analysis thematic synthesis critical interpretive synthesis
Santesso et al., 2021 (41)	NI	Social and patient perspective	Critical evaluation: CASP qualitative studies checklist, adapted version of the CASP tool (Atkins et al. 2008) Assess confidence of findings - GRADE- -CERQual - Confidence in the Evidence from Reviews of Qualitative research Present qualitative synthesis: thematic synthesis
SBU, 2016 (55)	*Qualitative research can be utilised to investigate a person’s perceptions, experiences, histories and interpretation of a certain phenomenon. It is also of value for disclosing potential barriers to change in a system and a person’s inclination/disinclination to undergo change.*	Individual perspective	Acronym – SPICE – Setting, Perspective, Intervention Comparison, Evaluation Critical evaluation: SBU’s check-list Present qualitative synthesis: SBU method
Scottish Intercollegiate Guidelines Network, 2019 (53)	NI	Patient perspective	NA
Scottish Medicines Consortium, 2022 (11)	NI	Thoughts, opinions, stories and feelings of patients and caregivers.	NI
Steigenberger et al., 2021 (48)	NI	Patient and social perspective	Framework- EUnetHTA HTA Core Model (Domain Patients and social aspects (SOC) Present qualitative synthesis: thematic synthesis
WHO, 2021 (60)	*A systematic review where primary qualitative studies are identified, appraised and synthesized in a systematic manner.*	Perception of the parties involved (stakeholders) about the problem and evidence about the effects, benefits and harms of opting for a policy	Acronym - SPIDER (Sample, Phenomenon of Interest, Design, Evaluation, Research type) Critical evaluation: CASP tool adapted; Present qualitative synthesis: Meta-ethnography Grounded theory Thematic synthesis Narrative synthesis Framework synthesis Critical interpretive synthesis Assess confidence of findings - GRADE- -CERQual - Confidence in the Evidence from Reviews of Qualitative research Present qualitative synthesis: Enhancing Transparency in Reporting the synthesis of Qualitative research (ENTREQ), eMERGe for reporting of meta-ethnographies, RAMESES for realist syntheses and meta-narrative reviews include reporting of decisions made on reasons why particular data were extracted, and STARLITE is used for reporting the literature search component of any QES.

Abbreviations: NI – Not informed, NA – Not applicable

The Qualitative evidence synthesis was defined by most authors as a method to integrate findings from qualitative in dividual studies/research, such as views, beliefs, experiences, and people’s perspectives.

We found that the most common justification for using qualitative data in HTA, independent of the author’s origin, incorporate information about patients’ or other stakeholders’ perspectives to identify acceptability, feasibility, and equity aspects.

The mapping identified several stages related to the synthesis of qualitative evidence: an acronym to guide the research question, instruments for assessing the quality of studies, methods for synthesizing evidence, instruments to guide study writing, a tool to assist in the decision-making of suitable methods for evidence synthesis, and a tool to assign a quality level to the evidence. Although information is available for all these stages, the analysis revealed that not all documents address every stage of the process; several documents present only one stage.

In the research question framework, SPICE was the most frequently endorsed strategy (43;52–54). RETREAT was the most cited framework for defining a method for synthesizing qualitative evidence (55;42–44). Thematic synthesis was used to synthesize evidence cited more times (7;36;38;53;54;45–48;56;57). The most cited instrument for assessing quality was CASP (Critical Appraisal Skills Program) (1;24;43;38;39;41;54;56;57). GRADE- CERQual was the main tool was to grade the quality of evidence (1;18;19;24;35;38–41;43;53;57). Enhancing Transparency in Reporting the synthesis of Qualitative research (ENTREQ) was a framework for reporting qualitative research cited with more frequence (43;57). In the topic recommendation, the GRADE evidence-to-decision (EtD) framework was a unique tool cited by five documents (39–41;43;53).

The compilation of the strategies informed by the documents according to the steps for conducting a synthesis of qualitative evidence is described in Table 3.

**Table 3.** Compilation of the strategies informed according to each step of the qualitative evidence synthesis process

Steps	Data
Research question framework	PerSPEcTiF (Perspective, Setting, Phenomenon of interest/problem, Environment (optional Comparison), Time/timing Findings) SPIDER (Sample, Phenomenon of Interest, Design, Evaluation, Research type) SPICE (Settings (S), Population/Perspective (P) Intervention (I), Comparator (C), Evaluation (E)) PICO (Population, Intervention, Comparator, Outcome)
Framework for defining method for synthesizing qualitative evidence	RETREAT (Research question, Epistemology, Time/Timeframe, Resources, Expertise, Audience & Purpose, Type of Data) framework - (“INTEGRATE- HTA”) EUnetHTA HTA Core Model (Domain Patients and social aspects (SOC) The Context and Implementation of Complex Interventions (CICI framework) NHS Patient Experience Framework Danish Centre for Health Technology Assessment HTA (DACHENTA) Handbook
Synthesizing methodology	Meta-ethnography, Grounded theory, Thematic synthesis, Narrative synthesis, Framework synthesis, Realist review or synthesis, Critical interpretive synthesis, meta-interpretation, meta-synthesis, Metanarrative, meta-study, ‘conceptual mapping’, meta-summary, qualitative cross-case analysis, Critical interpretive synthesis, SBU method, Best fit framework approach
Quality appraisal instrument for individual studies	CASP (Critical Appraisal Skills Program), ETQS, JBI, Popay, QF, ETQS, the Cochrane manual (Chapter 20), the cabinet office Quality in Qualitative Evaluation tool and Walsh, SBU’s check-list
Quality appraisal instrument for syntheses	GRADE- CERQual - Confidence in the Evidence from Reviews of Qualitative research
Reporting guidelines	Enhancing Transparency in Reporting the synthesis of Qualitative research (ENTREQ), COREQ, SRQR e MERGe for reporting of meta-ethnographies, RAMESES for realist syntheses and meta-narrative reviews include reporting of decisions made on reasons why particular data were extracted, and STARLITE is used for reporting the literature search component of any QES. Quality of Reporting Tool (QuaRT)
Recommendation	GRADE evidence-to-decision (EtD) framework (effectiveness, resource use, acceptability, feasibility, and equity)

## Discussion

According to our findings, some HTA agencies with more experience in the field (e.g., NICE, SMC, and SBU) include statements in their documents/guides related to the importance of considering qualitative data in the HTA process. However, an explanation of how to conduct the process was found only in three documents published by HTA agencies from Sweden, Scotland, and Germany (24;52;54).

The adoption of health technology assessments has grown in significance as countries allocate limited resources to maximizing patient health benefits (58).

Historically, more attention was given to costs and effectiveness as criteria for recommending technologies rather than social or ethical consequences or questions of acceptance (62).

Nevertheless, informed decisions require an interdisciplinary nature of HTA processes (54). From this perspective, the inclusion of qualitative data in HTA processes not only endorses the interdisciplinary nature of HTA analyses but also broadens the scope of health information that supports decision-making in determining evidence-based healthcare.

However, according to Germeni and Szabo (23), the integration of qualitative methodologies must align with established quality and reporting standards, concentrating on domains in which they can effectively illuminate issues that quantitative methods may not adequately capture. This ensures the realization of the full potential. They contend that fostering methodological innovation in the application of swift qualitative techniques and devising new strategies to leverage the synergy between qualitative and quantitative data in mixed methods research would greatly enhance the future of the HTA field. Regardless of the present mapping, researchers and HTA agencies have proposed methodological alternative frameworks, tools, and checklists to standardize the process.

Staniszewska et al. (45) highlighted that HTA agencies have made progress by including patient evidence in their assessments. For example, the authors mentioned the SBU, and its Handbook of Assessment of Methods in Health Care and Social Services, which includes chapters on patient-based evidence and the evaluation and synthesis of studies using qualitative methods. Only these two documents organize and present steps with specific methods, as a guide, while most of the documents analyzed in this review present separate suggestions for acronyms, frameworks, methods of synthesizing evidence, instruments to assess quality, tools to grade the quality of evidence, frameworks for reporting qualitative research, and recommendations.

Most findings of this review are similar to those of Sousa et al. (63) who published an introductory paper on qualitative evidence synthesis methodologies. The tools identified in this review were the Retreat framework, SPIDER, SPICE, and PerSPEcTiF. To describe the level of confidence in the evidence produced by a synthesis, GRADE CerQual was cited. Sousa et al. (63) reported similar tools for qualitative synthesis, such as ENTREQ and eMERGe (60).

Considering the reasons for incorporating qualitative data into HTA reports, the presented mapping of the most common justification was the patients’ or other stakeholders’ opinions and experiences to primarily identify aspects related to acceptability, feasibility, and equity. This finding is in accordance with that of Leys (59), who found that qualitative research can contribute to the HTA process by offering the perspectives, meanings, values, and interests of different stakeholders regarding technology. This reinforces the fact that qualitative research directs its attention to the social realm, equipping investigators with the means to explore health phenomena through the lenses of those who directly encounter them (64).

The structuring and standardization of processes can provide information to support decision-making by incorporating qualitative evidence into the HTA process and improving the quality of recommendations, providing evidence of feasibility, appropriateness, and significance, as well as patient values and preferences, acceptability, and equity. In addition, it can reduce methodological variations by allowing standardization of the process, making it easier to compare across different contexts while respecting the principles of transferability and equity.

### Strengths and limitations

This review was limited to discussing the frameworks for including the qualitative evidence in the HTA process; potentially missing are qualitative evidence synthesis methods that were not developed specifically or described in this context.

This mapping demonstrated that the field of HTA has expanded the way technologies are assessed, and HTA agencies have include frameworks to guide the inclusion of qualitative evidence. However, another important limitation is that this review did not analyze the reports from different agencies to identify the practical application of these tools/instruments (process and results).

### Implications for research

In light of this review, it may be relevant to conduct a qualitative study involving stakeholders (health technology practitioners, industry representatives, decision-makers, health technology researchers, qualitative data experts, qualitative evidence synthesis specialists, patients, and family representatives) to identify barriers and facilitators to implementing and analyzing qualitative evidence in the process of HTA.

### Implications for practice

The findings of this mapping identified guidelines or frameworks for synthesizing qualitative evidence for use in HTA. This result can offer practitioners the option of including qualitative evidence synthesis to obtain opinions, experiences, patient-centered approaches, and social perspectives in the HTA process. This implies development of an HTA that includes evidence of feasibility, acceptability, and new outcomes for health services that can influence interventions on equity and human rights.

## Conclusions

This review, which mapped and described the frameworks, tools, and processes used to include, synthesize, and evaluate the quality of qualitative evidence in the HTA context showed that SPICE was the most cited acronym and RETREAT was the preferred framework for synthesizing qualitative evidence. Thematic synthesis was the most frequently cited method for evidence synthesis and CASP was the most frequently mentioned instrument for quality assessment. The GRADE-CERQual was the primary tool for grading evidence quality, and ENTREQ was the most cited framework for reporting qualitative research synthesis.

This review confirmed the recent trend of including qualitative evidence in HTA documents. Although the documents cited common instruments, methods, or tools, they had different proposals, and only a few documents encompassed all the necessary steps in the process.

The implementation of a proposal to incorporate qualitative data into HTA processes requires strategies for the entire process, starting with identifying and characterizing the audience that will use the information, defining how to convey knowledge and guidance, anddefining the requirements and competencies required to incorporate, analyze, and synthesize qualitative data to support decision-making in the field of healthcare.

## Supporting information

Cardoso et al. supplementary material 1 Cardoso et al. supplementary material

Cardoso et al. supplementary material 2 Cardoso et al. supplementary material

## Supplementary material

The supplementary material for this article can be found at http://doi.org/10.1017/S0266462324000369.

## References

[1] O’Rourke B, Oortwijn W, Schuller T; **International Joint Task Group** . The new definition of health technology assessment: A milestone in international collaboration. Int J Technol Assess Health Care. 2020;36:187–190. 32398176 10.1017/S0266462320000215

[2] Booth A. [Internet] A methodological update on the use of qualitative evidence in health technology assessment. Decision Support Unit, ScHARR, University of Sheffield, United Kingdom, Sheffield; 2020. [Cited 2023 Apr 29] Available from:

[3] Flemming K, Booth A, Garside R, Tunçalp Ö, Noyes J. Qualitative evidence synthesis for complex interventions and guideline development: clarification of the purpose, designs and relevant methods. BMJ Glob Health. 2019;4(Suppl 1):e000882. 10.1136/bmjgh-2018-000882 PMC6350756 30775015

[4] Flemming K, Noyes J. Qualitative Evidence Synthesis: Where Are We at?. Int J Qual Methods. 2021;20:1–13.

[5] Lockwood C, Porritt K, Munn Z, Rittenmeye RL, Salmond S, Bjerrum M et al. [Internet] Chapter 2: Systematic reviews of qualitative evidence. In: Aromataris E., Munn Z. JBI Manual for Evidence Synthesis. Adelaide: JBI; 2020. [Cited 2023 Apr 29] Available from: https://jbi-global-wiki.refined.site/space/MANUAL/4688637/Chapter+2%3A+Systematic+reviews+of+qualitative+evidence

[6] Langlois EV, Tunçalp Ö, Norris SL, Askew I, Ghaffar A. Qualitative evidence to improve guidelines and health decision-making. Bull World Health Organ 2018;96(2):79–79A. 29403107 10.2471/BLT.17.206540 PMC5791783

[7] Carroll C. Qualitative evidence synthesis to improve implementation of clinical guidelines. BMJ 2017;356:j80. Published 2017 Jan 16. 28093384 10.1136/bmj.j80

[8] Brasil. [Internet] Diretrizes metodológicas: elaboração de revisão sistemática e meta-análise de ensaios clínicos randomizados. Ministério da Saúde, Secretaria de Ciência, Tecnologia, Inovação e Insumos Estratégicos em Saúde, Departamento de Gestão e Incorporação de Tecnologias em Saúde, Brasília, Ministério da Saúde; 2021 [Cited 2023 Apr 29] Available from: https://rebrats.saude.gov.br/phocadownload/diretrizes/20210622_Diretriz_Revisao_Sistematica_2021.pdf

[9] **National Institute for Health and Care Excellence** . [Internet] NICE health technology evaluations: the manual. United Kington; 2022. [Cited 2023 Jun 25] Available from: https://www.nice.org.uk/process/pmg36/resources/nice-health-technology-evaluations-the-manual-pdf-72286779244741

[10] CADTH. [Internet] Health Technology Review Rapid Review Process. Canada’s Drug and Health Technology Agency. Canada; 2022. [Cited 2023 Apr 29] Available from: https://www.cadth.ca/sites/default/files/attachments/2022-02/Rapid%20Review%20Process_0.pdf

[11] Scottish Intercollegiate Guidelines Network (SIGN). [Internet] SIGN 50: A guideline developer’s handbook. Edinburgh; 2019. [Cited 2023 Apr 29] Available from:http://www.sign.ac.uk https://www.sheffield.ac.uk/media/34045/download?attachment

[12] Pearson A, Robertson-Malt S, Rittenmeyer L. [Internet] Synthesizing Qualitative Evidence. Lippincott-Joanna Briggs Institute, Australia; 2011. [Cited 2023 Apr 29] Available from: https://nursing.lsuhsc.edu/JBI/docs/JBIBooks/Syn_Qual_Evidence.pdf

[13] Noyes J, Booth A, Cargo M, Flemming K, Harden A, Harris J et al. [Internet] Chapter 21: Qualitative evidence. In: Higgins JPT, Thomas J, Chandler J, Cumpston M, Li T, Page M et al. Cochrane Handbook for Systematic Reviews of Interventions version 6.2. London: Cochrane; 2021. [Cited 2023 Apr 29] Available from: https://training.cochrane.org/handbook/current/chapter-21

[14] Tong A, Flemming K, Mcinnes E, Oliver S, Craig J. Enhancing transparency in reporting the synthesis of qualitative research: ENTREQ. BMC Med Res Methodol. 2012;12(1):e181. 10.1186/1471-2288-12-181 PMC3552766 23185978

[15] Tong A, Sainsbury P, Craig J. Consolidated criteria for reporting qualitative research (COREQ): a 32-item checklist for interviews and focus groups. Int J Qual Health Care. 2007;19(6):349–357. 17872937 10.1093/intqhc/mzm042

[16] Staniszewska S, Simera I, Seers K, Mockford C, Goodlad S, Altman DG et al. GRIPP2 reporting checklists: tools to improve reporting of patient and public involvement in research. BMJ 2017;358:3453. 10.1136/bmj.j3453 PMC5539518 28768629

[17] Colvin J, Wainwright M, Noyes J, Munthe-Kaas H, Garside R, Carlsen B, et al. Confidence in the Evidence from Reviews of Qualitative research (CERQual): development and future directions of a novel approach. In: Chandler J, Mackenzie J, Boutron I, Welch V. (editors). Cochrane Methods. Cochrane Database of Systematic Review. 2015;1:45–47.

[18] Booth A, Noyes J, Flemming K, Gerhardus A, Wahlster P, van der Wilt GJ, Mozygemba K, Refolo P, Sacchini D, Tummers M, Rehfuess E. Structured methodology review identified seven (RETREAT) criteria for selecting qualitative evidence synthesis approaches. J Clin Epidemiol. 2018;99:41–52. 29548841 10.1016/j.jclinepi.2018.03.003

[19] Lewin S, Booth A, Glenton C, Munthe-Kaas H, Rashidian A, Wainwright M, et al. Applying GRADE-CERQual to qualitative evidence synthesis findings: introduction to the series. Implement Sci. 2018;13(1):2. 29384079 10.1186/s13012-017-0688-3 PMC5791040

[20] Lewin S, Glenton C, Munthe-Kaas H, Carlsen B, Colvin CJ, Gülmezoglu M et al. Using qualitative evidence in decision making for health and social interventions: an approach to assess confidence in findings from qualitative evidence syntheses (GRADE-CERQual). PLoS Med. 2015;12(10):001895. 10.1371/journal.pmed.1001895 PMC4624425 26506244

[21] Lockwood C, Munn Z, Porritt K. Qualitative research synthesis: methodological guidance for systematic reviewers utilizing meta-aggregation. Int J Evid Based Healthc. 2018;13(3):179–87. 10.1097/XEB.0000000000000062 26262565

[22] Noyes J, Booth A, Lewin S, Carlsen B, Glenton C, Colvin CJ et al. Applying GRADE-CERQual to qualitative evidence synthesis findings-paper 6: how to assess relevance of the data. Implement Sci. 2018;13(1):4. 29384080 10.1186/s13012-017-0693-6 PMC5791042

[23] Munn Z, Porritt K, Lockwood C, Person A. Establishing confidence in the output of qualitative research synthesis: the ConQual approach. BMC Med Res Methodol. 2014;14(108):1–7. 25927294 10.1186/1471-2288-14-108 PMC4190351

[24] Germeni E, Szabo S. Beyond clinical and cost-effectiveness: The contribution of qualitative research to health technology assessment. Int J Technol Assess Health Care. 2023;39(1)e23:1–4. 37092753 10.1017/S0266462323000211 PMC11570152

[25] Szabo SM, Hawkins NS, Germeni E The extent and quality of qualitative evidence included in health technology assessments: a review of submissions to NICE and CADTH. Int J Technol Assess Health Care 2024;40(1)e6:1–7. 10.1017/S0266462323002829 PMC10859830 38126273

[26] Wang YY, Liang DD, Lu C, Shi YX, Zhang J, Cao Y, et al. An exploration of how developers use qualitative evidence: content analysis and critical appraisal of guidelines. BMC Med Res Methodol 2020;20(1):160. 32552780 10.1186/s12874-020-01041-8 PMC7302150

[27] . Institut für Qualität und Wirtschaftlichkeit im Gesundheitswesen. [Internet] Allgemeine Methoden Entwurf für Version 7.0. IQWiG. Germany*;* 2022. [Cited 2023 Apr 29] Available from: https://www.iqwig.de/methoden/allgemeine-methoden_entwurf-fuer-version-7.pdf

[28] Murphy E, Dingwall R, Greatbatch D, Parker S, Watson P. Qualitative research methods in health technology assessment: a review of the literature. Health Technol Assess. 1999;2(16):iii–274. 9919458

[29] Peters MDJ, Godfrey C, McInerney P, Munn Z, Tricco AC, Khalil H. [Internet] Chapter 11: Scoping Reviews. In: Aromataris E, Munn Z (Editors). JBI Manual for Evidence Synthesis, JBI; 2020. [Cited 2023 Apr 29] Available from: https://jbi-global-wiki.refined.site/space/MANUAL/4687342/Chapter+11%3A+Scoping+reviews

[30] Tricco AC, Lillie E, Zarin W, O’Brien KK, Colquhoun H, Levac D, et al. PRISMA extension for scoping reviews (PRISMA-ScR): checklist and explanation. The PRISMA-ScR Statement. Ann Intern Med. 2018;169(7):467–73. 30178033 10.7326/M18-0850

[31] Marques RT, Machado-Rugolo J, Thabane L, Vantone M, Püschel VAA, Weber SAT, Cardoso MMA. Frameworks for Synthesizing Qualitative Evidence in Health Technology Assessment: A Scoping Review Protocol. Int J Qual Methods. 2023;22:11–7.

[32] McMeekin N, Wu O, Germeni E, Briggs A. How methodological frameworks are being developed: evidence from a scoping review. BMC Med Res Methodol 20, 173 (2020). 32605535 10.1186/s12874-020-01061-4 PMC7325096

[33] McKenzie JE, Brennan SE. [Internet] Chapter 12: Synthesizing and presenting findings using other methods. In: Higgins JPT, Thomas J, Chandler J, Cumpston M, Li T, Page MJ, Welch VA (editors). Cochrane Handbook for Systematic Reviews of Interventions version 6.4 (updated August 2023). Cochrane. 2023. [Cited 2023 Apr 29] Available from: www.training.cochrane.org/handbook

[34] Bertram M, Dhaene G, Tan-Torres ET. [Internet] Institutionalizing health technology assessment mechanisms: a how to guide. Geneva: World Health Organization; 2021. [Cited 2023 Apr 29] Available from: https://www.who.int/publications/i/item/9789240020665

[35] Page MJ, McKenzie JE, Bossuyt PM, Boutron I, Goffmann TC, Mulrow CD, et al. The PRISMA 2020 statement: an updated guideline for reporting systematic reviews. BMJ 2021;372:n71. 33782057 10.1136/bmj.n71 PMC8005924

[36] Levac D, Colquhoun H, O’Brien KK, Scoping studies: advancing the methodology. Implement 2010;20(5):69. 10.1186/1748-5908-5-69 PMC2954944 20854677

[37] Pollock D, Peters MDJ, Khalil H, et al. Recommendations for the extraction, analysis, and presentation of results in scoping reviews. JBI Evid Synth 2023;21(3):520–532. 36081365 10.11124/JBIES-22-00123

[38] Barreto JOM, Lewin S. Uso da evidência qualitativa para informar decisões no Brasil e na região da América Latina. Bis 2019;20(2):23–35.

[39] Campbell F, Weeks L, Booth A, Kaunelis D, Smith A. A scoping review found increasing examples of rapid qualitative evidence syntheses and no methodological guidance. J Clin Epidemiol 2019;115:160–171. 31229582 10.1016/j.jclinepi.2019.05.032

[40] Majid U, Vanstone M. Appraising Qualitative Research for Evidence Syntheses: A Compendium of Quality Appraisal Tools. Qual Health Res 2018;28(13): 2115–2131. 30047306 10.1177/1049732318785358

[41] Santesso N, Lytvyn L, Graham K, Cowl J, Knaapen L. *[Internet] How to include research on patient and public views in guidelines*; 2021. [Cited 2023 Apr 29] Available from: https://www.researchgate.net/publication/355047100_How_to_include_research_on_patient_and_public_views_in_guidelines.

[42] Downe S, Finlayson KW, Lawrie TA, Lewin SA, Glenton C, Rosenbaum S, Barreix M, Tunçalp Ö. Qualitative Evidence Synthesis (QES) for Guidelines: Paper 1 - Using qualitative evidence synthesis to inform guideline scope and develop qualitative findings statements. Health Res Policy Syst. 2019;17(1):76. 31391057 10.1186/s12961-019-0467-5 PMC6686511

[43] Glenton C, Lewin S, Lawrie TA, Barreix M, Downe S, Finlayson KW, Tamrat T, Rosenbaum S, Tunçalp Ö. Qualitative Evidence Synthesis (QES) for Guidelines: Paper 3 - Using qualitative evidence syntheses to develop implementation considerations and inform implementation processes. Health Res Policy Syst. 2019;17(1):74. 31391071 10.1186/s12961-019-0450-1 PMC6686245

[44] Lewin S, Glenton C, Lawrie TA, Downe S, Finlayson KW, Rosenbaum S, Barreix M, Tunçalp Ö. Qualitative Evidence Synthesis (QES) for Guidelines: Paper 2 - Using qualitative evidence synthesis findings to inform evidence-to-decision frameworks and recommendations. Health Res Policy Syst*;* 2019;17(1):75. 31391119 10.1186/s12961-019-0468-4 PMC6686513

[45] Booth A, Lewin S, Claire C, Munthe-Kaas H, Toews I, Noyes J, et al. GRADE-CERQual Coordinating Team. Applying GRADE-CERQual to qualitative evidence synthesis findings-paper 7: understanding the potential impacts of dissemination bias. Implement Sci. 2018;13(1):12. 29384076 10.1186/s13012-017-0694-5 PMC5791043

[46] Carmona C, Baxter S, Carroll C. Systematic review of the methodological literature for integrating qualitative evidence syntheses into health guideline development. Res Synth Methods. 2021;12(4):491–505. 33591605 10.1002/jrsm.1483

[47] Booth A. Chapter 15 Qualitative evidence synthesis. In: Facey, K., Ploug Hansen, H. and Single, A., (eds.) Patient Involvement in HealthTechnology Assessment. Adis. 2017:187–199.

[48] Steigenberger C, Schnell-Inderst P, Siebert U. [Internet] Integrating patients and social aspects into health technology assessment. In: Kohlhammer VW, author; Schildmann J, Buch C, Zerth J, Editors. Defining the Value of Medical Interventions: Normative and Empirical Challenges [Internet]. Stuttgart (DE): W. Kohlhammer GmbH; 2021. [Cited 2023 Apr 29] Available from:https://www.ncbi.nlm.nih.gov/books/NBK585096/36256801

[49] Ring N, Jepson R, Ritchie K. Methods of synthesizing qualitative research studies for health technology assessment. Int J Technol Assess Health Care. 2011;27(4), 384–390. 22004781 10.1017/S0266462311000389

[50] NICE. [Internet] *Methods for the development of NICE public health guid*ance (third edition). National Institute for Health and Care Excellence. UK; 2012. [Cited 2023 Jun 25] Available from: https://www.nice.org.uk/process/pmg4/resources/methods-for-the-development-of-nice-public-health-guidance-third-edition-pdf-2007967445701

[51] NICE . [Internet] Guide to the methods of technology appraisal 2013. National Institute for Health and Care Excellence. UK; 2013. [Cited 2023 Jun 25] Available from: https://www.nice.org.uk/process/pmg9/resources/guide-to-the-methods-of-technology-appraisal-2013-pdf-2007975843781 27905712

[52] **NICE** . [Internet] Interim methods guide for developing good practice guidance. National Institute for Health and Care Excellence. UK; 2014. [Cited 2023 Jun 25] Available from: https://www.nice.org.uk/process/pmg15/resources/interim-methods-guide-for-developing-good-practice-guidance-pdf-72286656632773. 28230953

[53] Scottish Medicines Consortium. [Internet] A Guide for Patient Group Partners. Healthcare Improvement Scotland; 2022. [Cited 2023 Apr 29] Available from: https://www.scottishmedicines.org.uk/media/6784/smc-guide-revised-march-2022-0-4.pdf.

[54] Pharmaceutical Benefits Advisory Committee. [Internet] Guidelines for preparing a submission to the Pharmaceutical Benefits Advisory Committee Version 5.0. Commonwealth of Australia as represented by the Department of Health; 2016. [Cited 2023 Jun 25] Available from: https://pbac.pbs.gov.au/content/information/files/pbac-guidelines-version-5.pdf

[55] SBU. [Internet] Evaluation and synthesis of studies using qualitative methods of analysis. Stockholm: Swedish Agency for Health Technology Assessment and Assessment of Social Services (SBU); 2016. [Cited 2023 Sep 12] Available from: https://studylib.net/doc/18147972/evaluation-and-synthesis-of-studies-using-qualitative-met

[56] Langlois EV, Daniels K, Akl EA. [Internet] Evidence synthesis for health policy and systems: a methods guide. Geneva: World Health Organization; Licence: CC BY-NC-SA 3.0 IGO; 2018. [Cited 2023 Apr 29] Available from: https://ahpsr.who.int/publications/i/item/2018-10-08-evidence-synthesis-for-health-policy-and-systems-a-methods-guide 33877749

[57] Healthcare Improvement Scotland. [Internet] A guide to conducting rapid qualitative evidence synthesis for health technology assessment. NHS Scotland; 2019 [Cited 2023 Nov 10] Available from: https://past.htai.org/wp-content/uploads/2019/11/Rapid-qualitative-evidence-synthesis-guide.pdf

[58] Booth A, Noyes J, Flemming K, Gerhardus A, Wahlster P, Van der wilt GJ, Mozygemba K, Refolo P, Sacchini D, Tummers M, Rehfuess E. [Internet] *Guidance on choosing qualitative evidence synthesis methods for use in health technology assessments of complex interventions.* 2016 [Cited 2023 Apr 29] Available from: http://www.integrate-hta.eu/downloads/

[59] Ring N, Ritchie K, Mandava L, Jepson R. [Internet] *A guide to synthesising qualitative research for researchers undertaking health technology assessments and systematic reviews*; 2010. [Cited 2023 Jul 11] Available from: http://www.nhshealthquality.org/nhsqis/8837.html

[60] WHO. Guide to qualitative evidence synthesis: evidence-informed policy-making using research in the EVIPNET framework. Copenhagen: WHO Regional Office for Europe. License: CC BY-NC-SA 3.0 IGO; 2021. [Cited 2023 Apr 29] Available from: https://www.who.int/publications/i/item/WHO-EURO-2021-2272-42027-57819

[61] Staniszewska S, Söderholm WS. Mind the evidence gap: the use of patient-based evidence to create “complete HTA” in the twenty-first century. Int J Technol Assess Health Care. 2021;37(e46):1–7. 10.1017/S026646232100012X 33745475

[62] Leys M. Health technology assessment: the contribution of qualitative research. Int J Technol Assess Health Care. 2003;19(2):317–329. 12862189 10.1017/s026646230300028x

[63] Sousa MSA, Wainwright M, Soares CB. Qualitative Evidence Synthesis: an introductory guide. BIS, Bol Inst Saúde 2019;20(2):7–22.

[64] Khankeh H, Ranjbar M, Khorasani-Zavareh D, Zargham-Boroujeni A, Johansson E. Challenges in conducting qualitative research in health: A conceptual paper. Iran J Nurs Midwifery Res 2015;20(6):635–41. 26793245 10.4103/1735-9066.170010 PMC4700679

[65] Pfadenhauer L, Rohwer A, Burns J, Booth A, Lysdahl KB, Hofmann B, Gerhardus A, Mozygemba K, Tummers M, Wahlster P, Rehfuess E. [Internet] *Guidance for the Assessment of Context and Implementation in Health Technology Assessments (HTA) and Systematic Reviews of Complex Interventions: The Context and Implementation of Complex Interventions (CICI)* Framework; 2016. [Cited 2023 Apr 29] Available from: http://www.integrate-hta.eu/downloads/PharmaceuticalBenefits Advisory Committee

