# Supplementary material for: Mapping frameworks for synthesizing qualitative evidence in health technology assessment
Source: Int J Technol Assess Health Care. 2024 Nov 13;40(1):e53. doi: 10.1017/S0266462324000369 (PMC11579671; doi:10.1017/S0266462324000369)
Supplement: Cardoso et al. supplementary material 1 — Cardoso et al. supplementary material [file S0266462324000369sup001.docx]

# Appendix I: Search strategy

Search conducted on 24 March, year 2023*.*

| **Search** | **Query** | **Records retrieved** |
| --- | --- | --- |
| PubMed | #1"qualitative research"[MeSH Terms] OR "qualitative study"[Title/Abstract] OR "qualitative studies"[Title/Abstract] OR "qualitative research method"[Title/Abstract] OR "qualitative research methods"[Title/Abstract]  #2 "biomedical technology assessment"[Title/Abstract] OR "health technology assessment"[Title/Abstract] OR "health technology assessments"[Title/Abstract] OR "technology assessment"[Title/Abstract] OR "technology assessments"[Title/Abstract] OR "guideline"[Title/Abstract]  #3"systematic reviews as topic"[MeSH Terms] OR "systematic review"[Title/Abstract] OR "meta synthesis"[Title/Abstract] OR "meta synthesis approach"[Title/Abstract] OR "meta synthesis findings"[Title/Abstract] OR "meta synthesis meta ethnography"[Title/Abstract] OR "meta synthesis method"[Title/Abstract] OR "meta synthesis process"[Title/Abstract] OR "meta synthesis review"[Title/Abstract] OR "meta synthesis study"[Title/Abstract] OR "meta synthesis techniques"[Title/Abstract] OR "meta synthesis theory"[Title/Abstract] OR "qualitative synthesis"[Title/Abstract] OR "qualitative synthesis analysis"[Title/Abstract] OR "qualitative synthesis approach"[Title/Abstract] OR "qualitative synthesis method"[Title/Abstract] OR "metasynthesis"[Title/Abstract] OR "metasynthesis approach"[Title/Abstract] OR "metasynthesis methods"[Title/Abstract] OR "meta ethnography"[Title/Abstract] OR "meta ethnography approach"[Title/Abstract] OR "meta aggregation"[Title/Abstract] OR "meta aggregation approach"[Title/Abstract] OR "meta aggregation method"[Title/Abstract] OR "meta aggregative"[Title/Abstract] OR "meta aggregation methodology"[Title/Abstract] OR "meta aggregative approach"[Title/Abstract] OR "meta aggregative synthesis"[Title/Abstract] | 64 |
| Web of Science | #1qualitative research OR qualitative study OR qualitative studies  #2biomedical technology assessment OR health technology assessment OR health technology assessments OR technology assessment OR technology assessments  #3systematic review OR meta synthesis OR meta ethnography OR qualitative synthesis OR metasynthesis OR meta aggregation OR meta aggregative | 886 |
| Cochrane library | #1qualitative research OR qualitative study OR qualitative studies  #2biomedical technology assessment OR health technology assessment OR health technology assessments OR technology assessment OR technology assessments  #3systematic review OR meta synthesis OR meta ethnography OR qualitative synthesis OR metasynthesis OR meta aggregation OR meta aggregative | 500 |
| EMBASE | #4 #1 AND #2 AND #3 21  #3 'systematic review'/exp OR 'meta ethnography'/exp 380,983  #2 'biomedical technology assessment'/exp 16,370  #1 'qualitative research'/exp 107,793 | 30 |
| Scopus | ( TITLE-ABS-KEY ( {qualitative research} OR {qualitative study} OR {qualitative studies} ) AND TITLE-ABS-KEY ( {biomedical technology assessment} OR {health technology assessment} OR {health technology assessments} OR {technology assessment} OR {technology assessments} ) AND TITLE-ABS-KEY ( {systematic review} OR {meta synthesis} OR {meta synthesis approach} OR {meta synthesis findings} OR {meta synthesis method} OR {meta synthesis process} OR {meta synthesis review} OR {meta synthesis study} OR {meta synthesis techniques} OR {meta synthesis theory} OR {qualitative synthesis} OR {qualitative synthesis analysis} OR {qualitative synthesis approach} OR {qualitative synthesis method} OR {metasynthesis} OR {metasynthesis approach} OR {metasynthesis methods} OR {meta ethnography} OR {meta ethnography approach} OR {meta aggregation} OR {meta aggregation approach} OR {meta aggregation method} OR {meta aggregative} OR {meta aggregation methodology} OR {meta aggregative approach} OR {meta aggregative synthesis} ) ) | 76 |
| PsycoINFO | Any Field: “qualitative research” OR “qualitative study” OR “qualitative studies” AND Any Field: “biomedical technology assessment” OR “health technology assessment” OR “health technology assessments” OR “technology assessment” OR “technology assessments” AND Any Field: “systematic review” OR “meta synthesis” OR “meta synthesis approach” OR “meta synthesis findings” OR “meta synthesis method” OR “meta synthesis process” OR “meta synthesis review” OR “meta synthesis study” OR “meta synthesis techniques” OR “meta synthesis theory” OR “qualitative synthesis” OR “qualitative synthesis analysis” OR “qualitative synthesis approach” OR “qualitative synthesis method” OR “metasynthesis” OR “metasynthesis approach” OR “metasynthesis methods” OR “meta ethnography” OR “meta ethnography approach” OR “meta aggregation” OR “meta aggregation approach” OR “meta aggregation method” OR “meta aggregative” OR “meta aggregation methodology” OR “meta aggregative approach” OR “meta aggregative synthesis” | 27 |
| CINAHL | Any Field: “qualitative research” OR “qualitative study” OR “qualitative studies” AND Any Field: “biomedical technology assessment” OR “health technology assessment” OR “health technology assessments” OR “technology assessment” OR “technology assessments” AND Any Field: “systematic review” OR “meta synthesis” OR “meta synthesis approach” OR “meta synthesis findings” OR “meta synthesis method” OR “meta synthesis process” OR “meta synthesis review” OR “meta synthesis study” OR “meta synthesis techniques” OR “meta synthesis theory” OR “qualitative synthesis” OR “qualitative synthesis analysis” OR “qualitative synthesis approach” OR “qualitative synthesis method” OR “metasynthesis” OR “metasynthesis approach” OR “metasynthesis methods” OR “meta ethnography” OR “meta ethnography approach” OR “meta aggregation” OR “meta aggregation approach” OR “meta aggregation method” OR “meta aggregative” OR “meta aggregation methodology” OR “meta aggregative approach” OR “meta aggregative synthesis” | 44 |
| BVS | (“qualitative research” OR “qualitative study” OR “qualitative studies”) AND (“biomedical technology assessment” OR “health technology assessment” OR “health technology assessments” OR “technology assessment” OR “technology assessments”) AND (“systematic review” ) | 73 |
| Science direct | (“qualitative research” OR “qualitative study” OR “qualitative studies”) AND (“biomedical technology assessment” OR “health technology assessment” OR “health technology assessments” OR “technology assessment” OR “technology assessments”) AND (“systematic review” ) | 481 |
| IJTAHC | qualitative | 5 |
| JBI | 1 (qualitative research or qualitative study or qualitative studies).mp. [mp=text, heading word, subject area node word, title] 1339  2 (biomedical technology assessment or health technology assessment or health technology assessments or technology assessment or technology assessments).mp. [mp=text, heading word, subject area node word, title] 156  3 (systematic review or meta synthesis or meta ethnography or qualitative synthesis or metasynthesis or meta aggregation or meta aggregative).mp. [mp=text, heading word, subject area node word, title] 5831  4 1 and 2 and 3 | 38 |
| PROQUEST | summary(“qualitative research” OR “qualitative study” OR “qualitative studies”) AND summary(“biomedical technology assessment” OR “health technology assessment” OR “health technology assessments” OR “technology assessment” OR “technology assessments”) AND summary(“systematic review” OR “meta synthesis” OR “meta synthesis approach” OR “meta synthesis findings” OR “meta synthesis method” OR “meta synthesis process” OR “meta synthesis review” OR “meta synthesis study” OR “meta synthesis techniques” OR “meta synthesis theory” OR “qualitative synthesis” OR “qualitative synthesis analysis” OR “qualitative synthesis approach” OR “qualitative synthesis method” OR “metasynthesis” OR “metasynthesis approach” OR “metasynthesis methods” OR “meta ethnography” OR “meta ethnography approach” OR “meta aggregation” OR “meta aggregation approach” OR “meta aggregation method” OR “meta aggregative” OR “meta aggregation methodology” OR “meta aggregative approach” OR “meta aggregative synthesis”) | 3 |
| Google scholar | “qualitative evidence” AND “health technology assessment” AND “systematic review” | 137 |
| HTA agencies | There is no specific strategy. The word “qualitative” was put in the search and all methods documents were analyzed. |  |
| Thesis databases and open grey | There is no specific strategy. The word “qualitative” was put in the search and all methods documents were analyzed. |  |
| No Limited period | | |
