# Supplementary material for: Mapping frameworks for synthesizing qualitative evidence in health technology assessment
Source: Int J Technol Assess Health Care. 2024 Nov 13;40(1):e53. doi: 10.1017/S0266462324000369 (PMC11579671; doi:10.1017/S0266462324000369)
Supplement: Cardoso et al. supplementary material 2 — Cardoso et al. supplementary material [file S0266462324000369sup002.docx]

Supplementary Table 1. Details about methods and tools, according to sources and region.

| **Methods, and tools** | **Region** | **Source** |
| --- | --- | --- |
| CASP (Critical Appraisal Skills Program) | Europe | <https://casp-uk.net/checklists/casp-qualitative-studies-checklist-fillable.pdf> |
| GRADE- CERQual | Multiregion | <https://www.cerqual.org/> |
| Enhancing Transparency in Reporting the synthesis of Qualitative research (ENTREQ) | Oceania | <https://www.ncbi.nlm.nih.gov/pmc/articles/PMC3552766/pdf/1471-2288-12-181.pdf> |
| GRADE evidence-to-decision (EtD) framework | Multiregion | <https://www.decide-collaboration.eu/evidence-decision-etd-framework> |
| RETREAT | Europe | Booth, A. , Noyes, J., Flemming, K. et al. (8 more authors) (2018) Structured methodology review identified seven (RETREAT) criteria for selecting qualitative evidence synthesis approaches. Journal of Clinical Epidemiology, 99. pp. 41-52. ISSN 0895-4356 |
| Cochrane qualitative checklist | Europe | <https://training.cochrane.org/handbook/current/chapter-21> |
| JBI checklist | Oceania | Lockwood C, Munn Z, Porritt K. Qualitative research synthesis: methodological guidance for systematic reviewers utilizing meta-aggregation. Int J Evid Based Healthc. 2015;13(3):179–187 |
| Cabinet Office checklist for social research | Europe | <https://assets.publishing.service.gov.uk/media/5a8179c1ed915d74e33fe69e/Quality-in-qualitative-evaulation_tcm6-38739.pdf> |
| PerSPEcTiF (Perspective, Setting, Phenomenon of interest/problem, Environment) | Europa e Asia | https://www.ncbi.nlm.nih.gov/pmc/articles/PMC6350737/ |
| INTEGRATE-HTA project | Europe | <https://www.cambridge.org/core/journals/international-journal-of-technology-assessment-in-health-care/article/abs/an-integrated-perspective-on-the-assessment-of-technologies-integratehta/66FF81AE93A7E0BD70940A0FCFF0A22E> |
| eMERGe for reporting of meta-ethnographies | Europe | France E.F., Cunningham, M., Ring, N. et al (2019). Improving reporting of meta-ethnography: the eMERGe reporting guidance. BMC Med Res Methodol 19, 25 |
| Report the synthesis of Qualitative research: Quality of Reporting Tool (QuaRT) | Europe | Carroll C, Booth A, Cooper K. A worked example of "best fit" framework synthesis: A systematic review of views concerning the taking of some potential chemopreventive agents, BMC Medical Research Methodology, 2011 11:29 |
| Popay | Europe | Popay J, Rogers A, Williams G. Rationale and Standards for the Systematic Review of Qualitative Literature in Health Services Research. Qualitative Health Research. 1998;8(3):341-351. doi:10.1177/104973239800800305 |
| Walsh | Europe | Walsh D, Downe S. Appraising the quality of qualitative research. Midwifery. 2006;22(2):108-119. doi:10.1016/j.midw.2005.05.004 |
| ETQS | Europe | Long AF, Godfrey M, Randall T, Brettle AJ and Grant MJ (2002) Developing Evidence Based Social Care Policy and Practice. Part 3: Feasibility of Undertaking Systematic Reviews in Social Care. Leeds: Nuffield Institute for Health. |
| COREQ | Oceania | Tong A, Sainsbury P, Craig J. Consolidated criteria for reporting qualitative research (COREQ): a 32-item checklist for interviews and focus groups. Int J Qual Health Care. 2007;19(6):349-357 |
| SRQR | North America | O'Brien BC, Harris IB, Beckman TJ, Reed DA, Cook DA. Standards for reporting qualitative research: a synthesis of recommendations. Acad Med. 2014;89(9):1245-1251. |
| Context and Implementation of Complex Interventions (CICI) framework | Europe | Pfadenhauer, L.M., Gerhardus, A., Mozygemba, K. et al. Making sense of complexity in context and implementation: the Context and Implementation of Complex Interventions (CICI) framework. Implementation Sci 12, 21 (2017). <https://doi.org/10.1186/s13012-017-0552-5> |
| EUnetHTA HTA Core Model | Europe | <https://www.eunethta.eu/hta-core-model/> |
| RAMESES | Oceania, Europe | Wong G, Greenhalgh T, Westhorp G, Pawson R. Development of methodological guidance, publication standards and training materials for realist and meta-narrative reviews: the RAMESES (Realist And Meta-narrative Evidence Syntheses – Evolving Standards) project. Southampton (UK): NIHR Journals Library; September 2014. |
| STARLITE | Europe | Booth A. "Brimful of STARLITE": toward standards for reporting literature searches. J Med Libr Assoc. 2006 Oct;94(4):421-9, e205. PMID: 17082834; PMCID: PMC1629442. |
| SPIDER | Europe | Cooke, A., Smith, D., & Booth, A. (2012). Beyond PICO: The SPIDER Tool for qualitative evidence synthesis. Qualitative Health Research, 22(10), 1435–1443. <https://doi.org/10.1177/1049732312452938> |
| SPICE | Europe | Booth, A. (2006). Clear and present questions: Formulating questions for evidence based practice. Library Hi Tech, 24(3), 355-368. <https://doi.org/10.1108/07378830610692127> |
| The Warwick Patient Experience Framework | Europe | Staniszewska S, Boardman F, Gunn L, et al. The Warwick Patient Experiences Framework: patient-based evidence in clinical guidelines. Int J Qual Health Care. 2014;26(2):151-157. doi:10.1093/intqhc/mzu003 |
| Danish Centre for Health Technology Assessment HTA (DACHENTA) Handbook | Europe | https://behandlingsraadet.dk/media/otjfhhzw/the-danish-health-technology-council-s-methods-guide-for-the-evaluation-of-health-technology.pdf |
